# Stem cell cultures derived from pediatric brain tumors accurately model the originating tumors

**DOI:** 10.18632/oncotarget.14826

**Published:** 2017-01-26

**Authors:** Anna Wenger, Susanna Larsson, Anna Danielsson, Kirstine Juul Elbæk, Petronella Kettunen, Magnus Tisell, Magnus Sabel, Birgitta Lannering, Claes Nordborg, Elizabeth Schepke, Helena Carén

**Affiliations:** ^1^ Department of Pathology, Sahlgrenska Cancer Center, Institute of Biomedicine, Sahlgrenska Academy, University of Gothenburg, Sweden; ^2^ Department of Oncology, Sahlgrenska Cancer Center, Institute of Clinical Sciences, Sahlgrenska Academy, University of Gothenburg, Sweden; ^3^ Department of Psychiatry and Neurochemistry, Institute of Neuroscience and Physiology, Sahlgrenska Academy, University of Gothenburg, Sweden; ^4^ Department of Neuropathology, Nuffield Department of Clinical Neurosciences, University of Oxford, John Radcliffe Hospital, Oxford, United Kingdom; ^5^ Department of Clinical Neuroscience and Rehabilitation, Institute of Neuroscience and Physiology, Sahlgrenska Academy, University of Gothenburg, Sweden; ^6^ Department of Pediatrics, Institute of Clinical Sciences, Sahlgrenska Academy, University of Gothenburg, Sweden; ^7^ The Queen Silvia Children’s Hospital, Sahlgrenska University Hospital, Gothenburg, Sweden; ^8^ Department of Pathology, Sahlgrenska University Hospital, Sweden

**Keywords:** DNA methylation, pediatric, glioblastoma, cancer stem cells, immunodeficient mice

## Abstract

Brain tumors are the leading cause of cancer-related death in children but high-grade gliomas in children and adolescents have remained a relatively under-investigated disease despite this. A better understanding of the cellular and molecular pathogenesis of the diseases is required in order to improve the outcome for these children. *In vitro*-cultured primary tumor cells from patients are indispensable tools for this purpose by enabling functional analyses and development of new therapies. However, relevant well-characterized *in vitro* cultures from pediatric gliomas cultured under serum-free conditions have been lacking. We have therefore established patient-derived *in vitro* cultures and performed thorough characterization of the cells using large-scale analyses of DNA methylation, copy-number alterations and investigated their stability during prolonged time in culture. We show that the cells were stable during prolonged culture in serum-free stem cell media without apparent alterations in morphology or growth rate. The cells were proliferative, positive for stem cell markers, able to respond to differentiation cues and initiated tumors in zebrafish and mice suggesting that the cells are cancer stem cells or progenitor cells. The cells accurately mirrored the tumor they were derived from in terms of methylation pattern, copy number alterations and DNA mutations. These unique primary *in vitro* cultures can thus be used as a relevant and robust model system for functional studies on pediatric brain tumors.

## INTRODUCTION

Brain tumors are the most common cause of cancer-related death in children and glioblastoma multiforme (GBM) is one of the most devastating forms [1]. Despite this, high-grade gliomas in children and adolescents have remained a relatively under-investigated disease. Recent genomic and epigenomic profiling studies have provided insight into the biology underlying these tumors in children which have also pointed to large differences compared to the tumors found in adults. One unique feature of pediatric GBM tumors is the presence of mutations in histone genes, at H3K27 and H3G34 [2–4]. Chromosomal copy number alterations (CNAs) are frequently found in adult GBMs with gains of chromosome 7 and loss of chromosome 10 being the most common (in 80-85% of cases) [5]. In pediatric cases, the number of chromosomal imbalances is generally lower, and ~15% of tumors lack any detectable CNAs [6].

High-grade brain tumors are thought to contain cancer stem cells, also called tumor-initiating cells or tumor propagating cells. Their presence in childhood brain tumors have been documented in medulloblastoma, GBM, diffuse intrinsic pontine glioma (DIPG) and ependymoma [7–11]. These cells are thought to withstand chemo and radiation therapy and enable tumor regrowth, even after an initially successful treatment [12].

Improving the outcome of children with brain tumors both in respect to survival and avoiding damage to the developing brain during treatment requires a better understanding of the cellular and molecular pathogenesis of the respective diseases. *In vitro*-cultured primary tumor cells from patients are indispensable tools for functional analyses and development of new therapies. However, appropriate well-characterized patient-derived *in vitro* cultures from pediatric high-grade gliomas are rare, reviewed by Xu et al [13]. The few published cell lines that are available are grown with serum [14–17] which is known to induce alterations to the cells [18]. We have therefore established patient-derived *in vitro* cultures grown under serum-free conditions, enriching for cells with stem cell properties, and performed thorough characterization of the cells using large-scale analyses of DNA methylation and CNAs as well as determined their stem cell properties and the genomic stability of the cells during prolonged time in culture.

In summary, we show that the cells can be maintained long-term in culture, retain the methylation profiles of the tumors they were generated from, are positive for stem cell markers, respond to differentiation treatment and are tumor-initiating when injected orthotopically in immunocompromised mice and zebrafish. The patient-derived *in vitro* cultures thus represent an applicable model system which will enable further functional analyses and enhance our knowledge about pediatric brain tumors.

## RESULTS

### Characteristics of primary tumors

**Figure 1:**
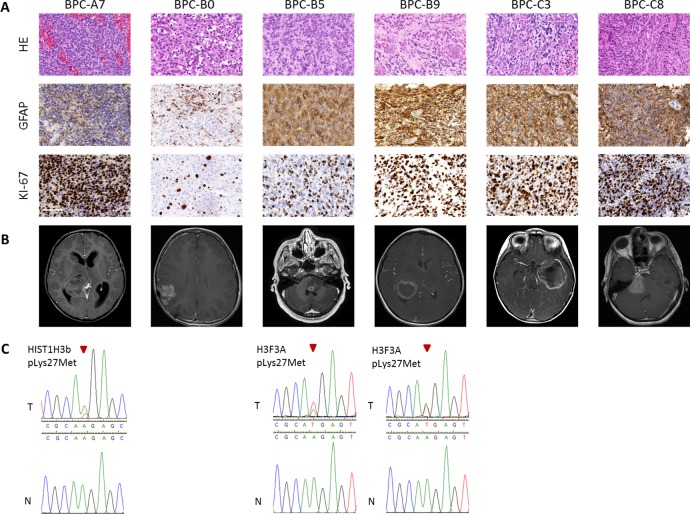
Characteristics of tumors **A**. Immunohistochemical analyses. H&E; GFAP, astrocytic marker; KI-67, proliferation marker. For the recurrent tumor BPC-B0, GFAP and KI-67 shown for the primary tumor but HE from recurrence. Scale bars, 100 μm; **B**. MRI scans of the patients, T1 weighted with gadolinium and **C**. Sanger sequencing of histone mutations in tumors (T; wildtype denoted as n).

**Figure 2:**
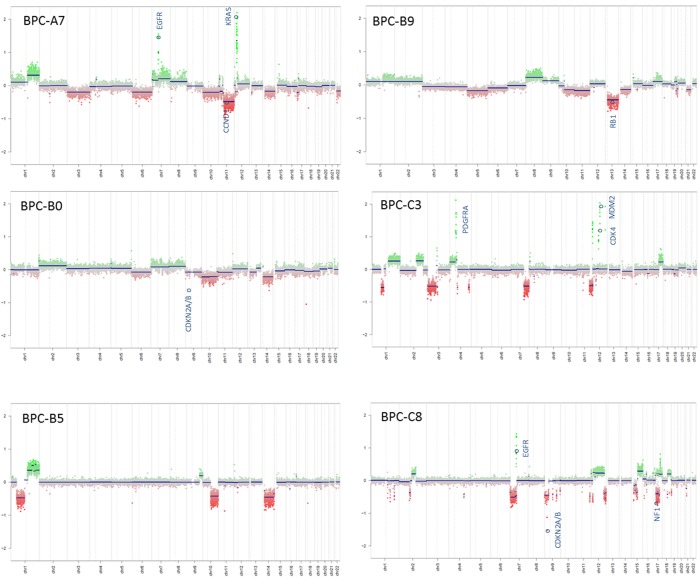
Copy-number alterations in the tumors The intensity values from Illumina 450K methylation arrays were used to generate CNA plots.

Tumor samples from six pediatric high-grade brain tumor patients were used in the study. The tumors were originally diagnosed as GBMs, CNS-Primitive neuroectodermal tumors (PNETs) or atypical teratoid/rhabdoid tumors (AT/RTs). Our MethPed classifier [19] using methylation profiles classified them all as GBMs and after review by a senior neuropathologist the samples were also histologically classified as GBMs. For patient data, see Table S1. The immunohistochemistry analyses that were used for diagnosis were obtained from the Pathology department at the Sahlgrenska University Hospital and MRI scans from the Radiology department (Figure 1A-1B, Supplementary Figure S1A-S1B). Imprints were made from all tumors used in the study and they were stained with hematoxylin and eosin (H&E). Tumor content was estimated by a senior neuropathologist to near 100% in all cases (Supplementary Figure S1B). We performed mutation screening of the genes H3 histone, family 3A (*H3F3A)*, histone cluster 1, H3b (*HIST1H3B)* and isocitrate dehydrogenase 1 (*IDH1*) and *IDH2*. Mutations in H3K27 were found in the tumors BPC-B5 and BPC-B9 and in *HIST1H3B* (K27) in BPC-A7 (Figure 1C). None of the samples had mutations in the *IDH* genes. The O-6-methylguanine-DNA methyltransferase (*MGMT*) gene was unmethylated (analyzed with the Illumina 450K methylation arrays and the R package mgmtstp27) in all cases. Using the intensity values from Illumina 450K methylation arrays we profiled CNAs in the primary tumors. The samples showed both segmental and structural aberrations (Figure 2) such as gain of chromosome 7, loss of chromosomes 10, 13 and 14; epidermal growth factor receptor (*EGFR*) and platelet-derived growth factor receptor, alpha polypeptide (*PDGFRA*) amplifications and deletions of retinoblastoma 1 (*RB1*), cyclin-dependent kinase inhibitor 2A/B (*CDKN2A/B*) and phosphatase and tensin homolog (*PTEN*).

### Pediatric CNS tumor cells can be cultured and maintained *in vitro*

**Figure 3:**
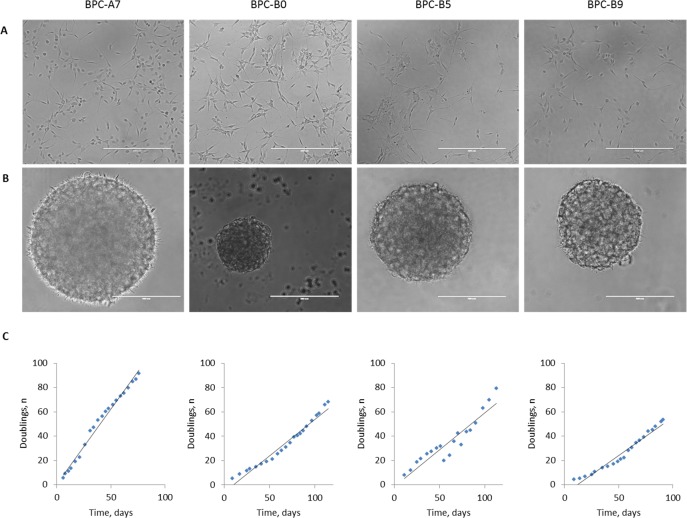
Morphology and growth rate of cultured cells **A**. Bright field images of four of the established *in vitro* cultures grown under adherent conditions; **B**. as tumor spheres and **C**. doubling rate of the adherent cells during 20 passages in culture.

Fresh tumor samples obtained from the neurosurgery operating theatre at the Sahlgrenska University Hospital were dissociated and cultured in neural stem cell media according to Pollard *et al*, [20] with some minor adjustments. Cultures were initiated with either the growth factors Fibroblast Growth Factor 2 (FGF-2) and Epidermal Growth Factor (EGF) or with EGF alone. We noted that some of the cultures proliferated faster with only EGF and some with EGF and FGF-2. Cells were cultured adherently on laminin-coated plastics (Figure 3A) but were also tested for tumor sphere formation (Figure 3B). The cells could be maintained long-term in culture as adherent cultures with no alterations in growth rate (Figure 3C). We also confirmed that the chromosomal profiles were largely maintained in the cultured cells (Supplementary Figure S2A), but differences do occur; the BPC-B5 tumor had a loss of part of chromosome 10 while the cultured cells had an intact chromosome 10, and all cell lines accumulated more alterations in high passage (P30, data not shown). The histone mutations identified in the tumors were present also in the cell cultures (Supplementary Figure S2B). BPC-A7 and BPC-B5 lost the wild-type allele in culture whereas BPC-B9 retained it.

The stability of the DNA content of the tumor cells was confirmed for all cell cultures using flow cytometry (FCM) analysis at different passages (Supplementary Figure S3A) and was found to be stable for all cultures. DNA histograms for BPC-A7 indicated a DNA index of 1.9, corresponding to near tetraploidy (diploid cells have a DNA index of 1.0), which was stable through repeated measurements and over time (passage 9-22) (Supplementary Figure S3A). The chromosome number analysis of methaphase chromosomes (*n* = 10) indicated that the cells were hypotetraploid with chromosome numbers of 76-82 in the cell line.

As it is known that *EGFR*-amplified GBM cells lose the *EGFR* amplification after a few passages in culture we studied this region in detail [21]. Two of the tumors harbored *EGFR* amplification; BPC-A7 and BPC-C8. Both cultures had the amplification retained after five passages in culture (Supplementary Figure S2A) but at passage 15 it was lost (data not shown). As it is likely that the amplification is lost in culture due to the high level of EGF that is supplemented in the media, we cultured the cells in media supplemented with FGF-2 instead of EGF and analyzed *EGFR* status with fluorescence *in situ* hybridization (FISH) analysis (Supplementary Figure S3B). Amplification of *EGFR* was verified in single cells from tumor imprint specimen and in cells after 6 passages of adherent culture in EGF supplemented media (Supplementary Figure S3B). These amplifications were present as clusters of nuclear signals with high-level DNA copy number gain in the tumor cells. It is thus likely that the *EGFR* gene copies in the tumor cells are present as extrachromosomal double minute (DM)-type micronuclei. The number of *EGFR* gene amplifications in these micronuclei seems to be diluted or lost during the propagation of cells in the presence of EGF but in contrast, *EGFR* amplification was retained after 15 passages in culture when cultured in only FGF-2 (Supplementary Figure S3B).

### Pediatric CNS tumor cells expanded in stem cell conditions are positive for neural stem cell markers and remain stable during culturing

**Figure 4:**
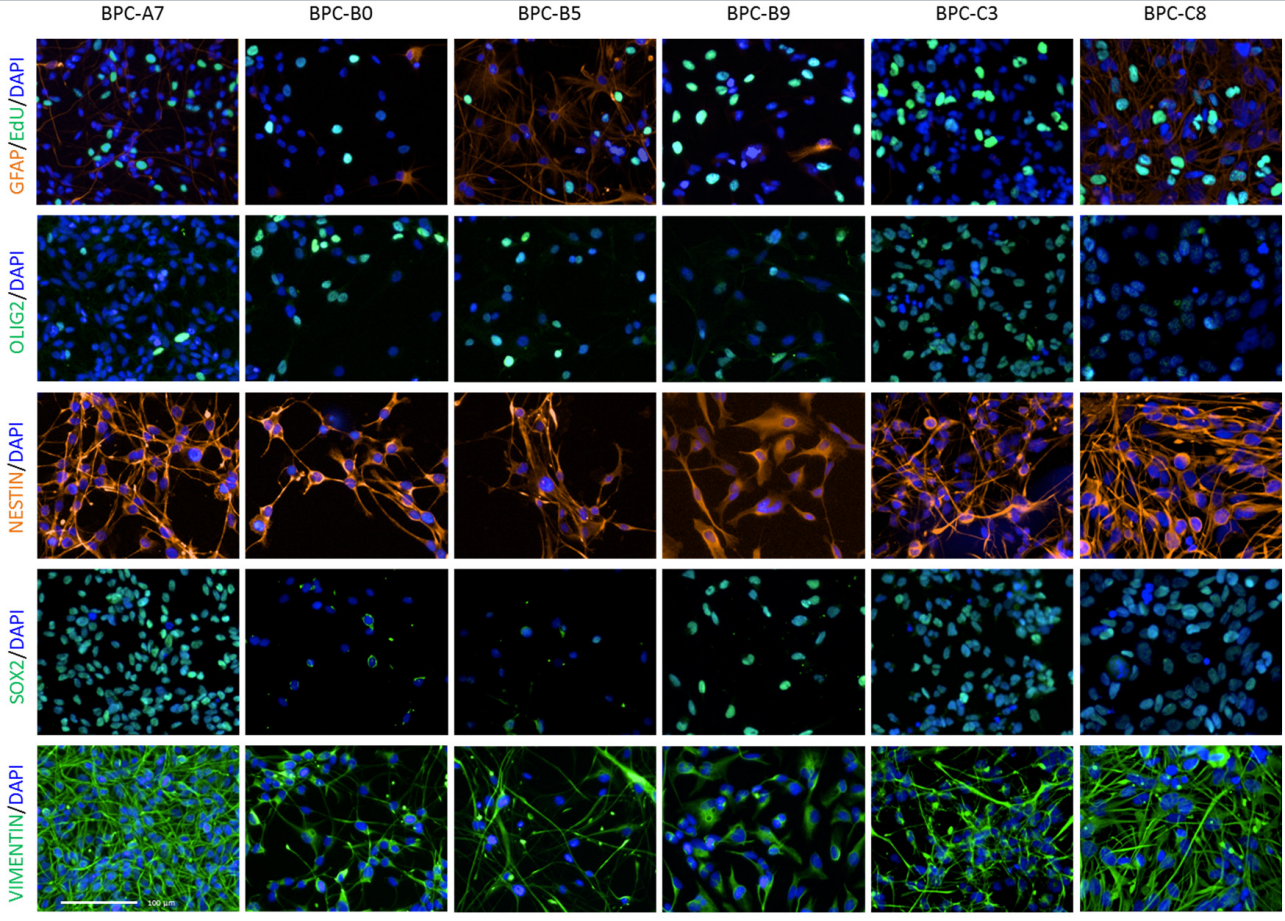
Protein expression in cultured cells Adherent cells labelled with DAPI (nuclear staining), EdU (proliferation marker), GFAP (astrocyte marker) and neural stem/progenitor cell markers OLIG2, nestin, SOX2 and vimentin. Scale bars, 100 μm.

We used immunocytochemistry to verify that the cells expressed stem cell/neural progenitor markers such as nestin, oligodendrocyte transcription factor 2 (OLIG2), SRY (sex determining region Y)-box 2 (SOX2) and vimentin. All cultures were highly positive for nestin and vimentin and were proliferative as measured by 5-ethynyl-2-deoxyuridine (EdU) incorporation (Figure 4). The frequency of OLIG2-positive cells varied in the different tumor cultures as did the expression of SOX2. BPC-B0, BPC-B5 and BPC-C8 were > 40% positive for SOX2 while the other cell lines (BPC-A7, BPC-B9 and BPC-C3) were more than 85% positive. Most cultures had low expression of the astrocyte marker glial fibrillary acidic protein (GFAP; Figure 4) and the neuronal marker microtubule-associated protein 2 (MAP2; data not shown). We also verified the same pattern for nestin and GFAP for the cell lines when they formed tumor spheres (Supplementary Figure S4).

### *In vitro* cultures preserve and maintain their methylation profile during extended time in culture

**Figure 5:**
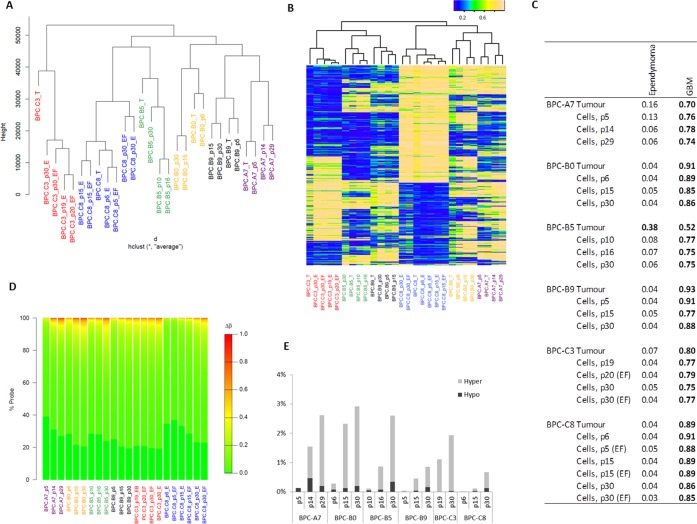
DNA methylation profile in cultured cells and their correlation to the primary tumor **A**. Dendrogram showing hierarchical clustering of all the sites on the methylation array; **B**. Unsupervised clustering of the top 5% of variable methylation sites; **C**. MethPed analysis demonstrates that the tumor class is maintained in cultured cells; **D**. For each sample, the absolute difference in β-value in the tumor compared to the cells is calculated at each probe and binned into 1% methylation difference increments (described by the color scale); the percentage of probes showing each methylation difference level is shown in the individual plots and **E**. percentage of sites with more than 0.51 difference in Δβ.

DNA methylation is an increasingly important tool when it comes to tumor classification [19] and has been shown to play an important role in pediatric brain tumors [22]. Previous studies have shown that tumors, briefly cultured primary cells and cell lines have different methylation profiles and cluster separately [23, 24]. We therefore profiled the methylation pattern in the primary tumors and corresponding *in vitro* cultures at different passages over a 30-passage time-course to evaluate how similar the cells are the originating tumor and thus how relevant the cultures are for studying the disease. We used the Illumina 450K methylation arrays and clustered the samples based on all probes (Figure 5A) and on the top 5% of CpGs with the most variable methylation (Figure 5B). The cultured cells clustered closely together with the primary tumor they were derived from, also at later passages, showing that the cells are not considerably altered in our culture system.

We next used our recently developed MethPed brain tumor classifier [19] to investigate if the cultured cells retain the original brain tumor classification (Figure 5C). All tumors showed a strong correlation with the GBM group of tumors which was preserved and maintained during culturing of the cells. For one of the samples, BPC-B5, the signature was increased for the cells in the culture compared to the primary tumor.

Culturing the cells in only EGF or in EGF + FGF-2 did not change the methylation profiles of the cells. Pearson correlation between the culture BPC-C3 cultured for 20 passages with EGF only or EGF + FGF was 1.0, the same correlation as for the culture BPC-C8 grown for 15 passages.

We next analyzed the number of sites where methylation was altered during culture and could conclude that very few of the investigated ~450 000 methylation sites were altered in the cultures (global methylation levels were not evaluated in this study), but the number increased with time in culture (Figure 5D). It has previously been shown that 95% of fully unmethylated probes display methylation β-values ≤ 0.31, while fully methylated probes have β-values ≥ 0.82; thus a Δβ threshold of 0.51 can be used as the minimum change expected for a CpG to be considered as going from fully unmethylated to methylated or vice versa [25, 26]. We therefore also explored the percentage of sites that altered with a Δβ of at least 0.51. This analysis showed that there were few sites that reverse methylation state; less than 0.3% in passage 5 cells and less than 3% at 30 passages (Figure 5E). The affected sites seemed to be largely random; the six cell lines only had 12 CpG sites in common that were altered in passage 30 cells compared to the tumors.

### Pediatric CNS *in vitro* cultures respond to differentiation cues

**Figure 6:**
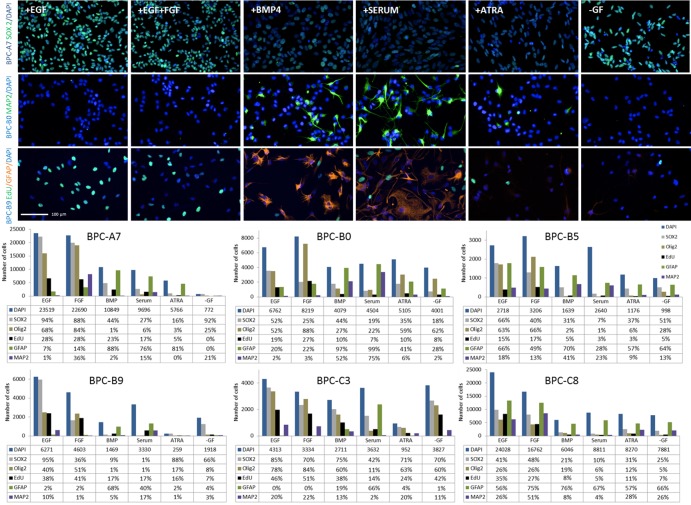
Response to differentiation cues in the different cell lines (Top panel) Immunocytochemistry of SOX2 (top row), MAP2 (second row) and EdU and GFAP (third row) for selected cell lines during normal culture conditions and exposed to differentiating conditions (BMP, Serum, ATRA and growth factor withdrawal) and (bottom) quantification of cells expressing different markers under these conditions. Cell numbers were normalized to the seeding density of the control (EGF). The total number of cells in each condition is presented as the median value and the number of positive cells for each marker in proportion to that. Note the difference in scale on the graphs. Scale bars, 100 μm.

As one feature of stem cells is their ability to differentiate, we induced the cells to differentiate in the presence of various differentiating compounds (Bone morphogenic protein 4 (BMP-4), serum, All-trans retinoic acid (ATRA) [27] [28] and growth factor withdrawal). All cell lines showed reduced proliferation, reduced expression of the stem cell markers OLIG2 (except BPC-B0) and SOX2 and upregulation of the astrocytic marker GFAP (except BPC-B5) and the neuronal marker MAP2 (at least for one of the differentiating compounds) compared to normal culture conditions (EGF; Figure 6). There were also cell line-specific responses; for example, FGF-2 induced differentiation of BPC-A7 but not the other cell cultures, for which it instead increased the proliferation (BPC-B0 and BPC-B5) or had little effect.

### The patient-derived *in vitro* cultures are suitable for high content screening assays

To analyze the reproducibility of the cells in drug screening assays we seeded cells in 96-well plates and treated them with the commonly used therapeutic agents Temozolomide, Etoposide and Vincristine. The response to the drug treatment varied greatly between the cell lines (Supplementary Figure S5). Both cells in untreated and treated wells showed very reproducible results with small error bars.

### The tumor cells are tumor-initiating when injected into zebrafish and immunodeficient mice

**Figure 7:**
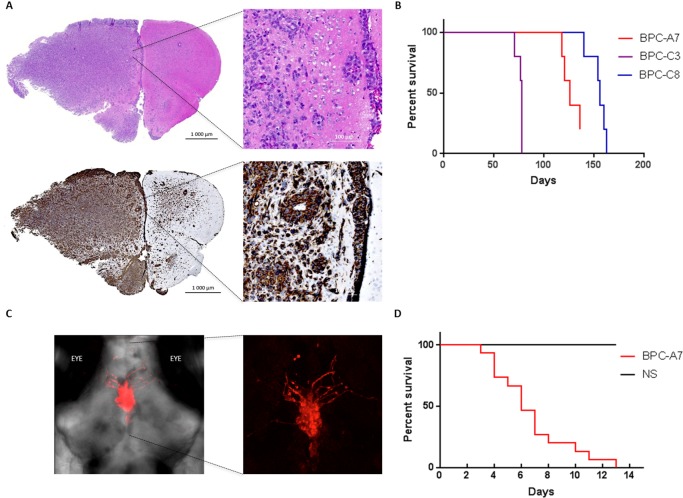
The pediatric tumor cells are tumorigenic when injected into the brains of immunodeficient mice and zebrafish **A**. Mouse brain orthotopically injected with BPC-C8 (1*10^5^ cells), stained with H&E (top) and human nestin (bottom) show massive infiltration of the injected hemisphere. **B**. Survival curve of all cell lines injected in mice; BPC-A7, BPC-C3 and BPC-C8. **C**. RFP-labelled BPC-A7 cells (left), enlarged picture (right), injected into the brain of two days old zebrafish show invasive growth into the brain. **D**. Survival curve of adult zebrafish injected with BPC-A7 and neural stem (NS) cells.

We next profiled the ability of the cells to give rise to tumors *in vivo* in immunodeficient mice and zebrafish. Three of the primary cell cultures; BPC-A7, BPC-C3 and BPC-C8 were orthtopically injected into mice and all three cell lines initiated tumor-growth. Mice were sacrificed at endpoint and H&E staining demonstrated a histological difference between tumor-invaded and non-invaded mouse brain. Staining for human nestin (hNestin) showed a very infiltrative tumor growth with onset in the injected cerebral hemisphere. Tumors grew in typical glioma patterns, so called Scherer's structures [29] where tumor cells surround nerve cells (Supplementary Figure S6A), blood vessels and show subpial accumulation (Figure 7A). Tumor cells invaded the white matter of the non-injected hemisphere (Supplementary Figure S6B). Survival curves for mice in all three groups exhibit a close time of death within the groups suggesting that each cell line is homogenous and have a similar growth rate *in vivo* (Figure 7B).

As zebrafish has been used for orthotopically injection of pediatric brain tumors [30], we also injected Red fluorescence protein (RFP)-labelled BPC-A7 cells into the brains of larval zebrafish. Tumor growth was monitored by confocal microscopy which revealed an invasive and aggressive growth pattern (Figure 7C). We additionally performed survival curves to compare adult zebrafish orthotopically injected with BPC-A7 tumor cells and normal neural stem (NS) cells respectively. The zebrafish injected with BPC-A7 all died within fourteen days, while the NS-injected fish survived during this time (Figure 7D).

## DISCUSSION

Brain tumors are the most frequent solid tumors among children. Despite this, the number of cases per year is low compared to in adults. The limited number of cases has led to a lack of knowledge about pediatric brain tumors compared to adults for whom large numbers of samples are easily accessible and relevant model systems exist [31] [20]. There is therefore a need for accurate model systems to enable functional studies on pediatric brain tumors. Additionally, clinical trials of new therapy often take many years to enroll enough patients and the number of trials available for this small group of patients is limited, which is why an accurate model system also for drug screenings is needed. The lack of pediatric brain tumor *in vitro* cultures is largely explained by the low number of available samples and the difficulty in culturing the cells under conditions that maintain the properties of the tumor cells. Therefore, we have developed culture conditions that enable their culturing under stem cell conditions in serum-free media.

In this study, we obtained six pediatric high-grade glioma tumors directly from surgery and established *in vitro* cultures from them. The tumors were all classified as GBMs by our methylation-based classifier, MethPed [19], and by review of a senior neuropathologist. The cells were cultured under stem cell enriching conditions and could be maintained in culture long-term ( > 30 passages) without apparent alterations in growth rate or morphology. The primary tumors had CNAs representative of the disease and most CNAs were maintained also in the cultured cells but alterations did occur. Differences between tumors and cell lines can be expected as the cells represent a homogenous population of stem cells while the primary tumor also contains “bulk cells” and cells of varying differentiation status. It is likely that these bulk cells contain the loss of part of chromosome 10 in the BPC-B5 tumor since the stem cells in culture have an intact chromosome 10. The increased GBM signature classification by MethPed for BPC-B5 cells compared to the tumor suggests a more homogeneous population of stem cells in culture with a selection for the histone mutation since the wild-type allele was lost in culture.

As previously known [21], *EGFR*-amplified cells lose their amplification rapidly in culture. Culturing *EGFR*-amplified GBM cells under our conditions, supplemented with only EGF, retained the amplification for at least five passages (but were lost after 15). By instead culturing the cells in FGF-2 (no EGF) we show that the amplification was retained at passage 15 and thus constitute an appropriate model system to study *EGFR*-amplified pediatric GBM tumor cells. Mutations in the histone genes have been shown to be a common feature in pediatric high-grade gliomas [2, 4]. Three of the tumors and the corresponding cultures in our study were mutated in either *H3F3A* or *HIST1H3B*, enabling in detail studies of these particular groups of pediatric GBMs with very distinct molecular profiles compared to wild-type GBMs. There is of course a necessity of developing specific therapies for mutated and histone wild-type tumors, as these forms are so markedly different.

The cells were positive for stem cell markers such as nestin and SOX2 and neural progenitor marker vimentin [32]. When induced to differentiate, the cell cultures showed various responses; most upregulated the astrocyte marker GFAP and the neuronal marker MAP2 and downregulated the stem cell/progenitor markers SOX2 and OLIG2. Some cultures showed a reduction in growth rate and proliferation while others did not have such a marked response. This points to the inter-patient heterogeneity of these tumors and the importance of identifying the correct treatment regime for each single patient, based on the properties of that specific tumor, which is further demonstrated by the dissimilar response of the cell lines to treatment with chemotherapeutic agents in our *in vitro* drug assay.

Methylation profiling can be used to diagnose and subgroup pediatric brain tumors [2, 4, 19, 33, 34] and the functional role of epigenetic alterations in brain tumors now needs to be uncovered. We therefore investigated the stability of the methylation state during culturing and compared to the primary tumors. There is a lack of publications reporting on methylation profiling of primary tumors and corresponding primary *in vitro* cultures. In a study on head and neck squamous cell carcinoma the authors concluded that there are many differences in the methylation profiles between cell cultures and originating tumor so we were interested in documenting alterations in our models [35]. However, in our system, the cultured cells accurately mirrored the tumors that they arose from, delineating differences in tumor type, adult *versus* pediatric origin and potentially also the need of optimal culture conditions for the cells to maintain their methylation profiles in culture.

Our thorough characterization shows that the primary cells are very stable in culture and tumor-initiating in mice and zebrafish with invasive growth pattern and forming Scherer's structures in mice thus mimicking human glioma. This allows for an accurate *in vitro* and *in vivo* model to study pediatric high-grade gliomas. Importantly, this model makes it possible to avoid the use of established cell lines that have been in culture for too long and lost many of the features of the tumor they originated from, which is an important step to ensure reliable and reproducible results.

In summary, we have established *in vitro* cultures from six high-grade pediatric brain tumors. All the primary cultures could be maintained in culture long-term without apparent alterations in growth rate or morphology. The cells reflected the primary tumors they originated from when it came to methylation profiling, CNAs and common DNA mutations. The cells were positive for stem cell markers, able to respond to differentiation cues and generated tumors similar to human glioma when orthotopically injected into zebrafish and mice. Together, these findings suggest that the cells are cancer stem cells. Furthermore we display the importance of serum-free media conditions and strict culture procedures to retain the features of the originating cells. These unique primary *in vitro* cultures that accurately mirror the originating tumors now enable important functional studies on pediatric high-grade brain tumors.

## MATERIALS AND METHODS

### Patients and samples

The study was approved by the Regional Ethical Review Board of Gothenburg (Dnr 604-12) and was carried out in accordance with the relevant guidelines and regulations. Tumor samples were obtained after signed informed consent from the parents of children who underwent surgery at the Sahlgrenska University Hospital. Samples were collected during 2013-2015. For patient data, see Table S1.

### Cell culturing

Tumor samples were dissociated into single cells using Accutase (Gibco) for 15 minutes at 37°C and the cell suspensions were passed through a 70 μM cell strainer and plated onto laminin (Sigma)-coated plastics in stem cell media (DMEM-F12 supplemented with B27 (Gibco), N2 (Gibco), EGF (10 ng/ml, Peprotech) and in some cases FGF-2 (10 ng/ml). Fresh media was added every fourth day and cells were passaged typically every week, 1:2-1:5, using Accutase. All established cell cultures were confirmed negative for mycoplasma contamination.

### Cellular analyses

#### Proliferation

EdU incorporation assays were performed using the Click-iT^®^ EdU Alexa Fluor^®^ 488 Imaging Kit (Invitrogen, Carlsbad, CA). Cells were grown in 96-well plates and pulsed with EdU for 24 hours prior to fixation in 4% paraformaldehyde (PFA).

#### Flow cytometric analysis of cell cycle phase distribution and identification of ploidy patterns

Cells were detached with Accutase solution and isolated nuclei were stained for 30 minutes with propidium iodide staining solution containing 0.1% Triton X-100, 10 μg/mL propidium iodide, and 100 μg/mL DNase-free RNase A in PBS (all Sigma-Aldrich). Chicken and rainbow trout red blood cells were co-stained in each sample and used as internal controls for DNA amount. Chicken red blood cells have a DNA content of 35% of human diploid cells while rainbow trout red blood cells have 80% of human diploid cells [36]. The distributions of G1, S, and G2 cells were analyzed at 72 hours after seeding using the FACScalibur system (BD Biosciences). The ModFit LT™ software (Verity Software House) was used for automatic analysis and peak detection to identify ploidy patterns.

#### Chromosome preparations and numerical chromosome aberrations analysis

Chromosome preparations for cytogenetic analysis were made as previously described [37]. Briefly, colcemid (0.05 g/mL, GibcoBRL, Life Technologies, NY) was added to the tumor cell cultures during the final 60 minutes before harvest. The cells were harvested by mitotic shake-off, pelleted by centrifugation, and resuspended in 0.075 M KCl at room temperature for 30 minutes. This was followed by a gradient of methanol-acetic acid fixation series from 9:1 to 3:1 (v/v ratio of methanol to acetic acid). Chromosomes were mounted with Prolong Antifade mounting medium with DAPI (Molecular Probes). The tumor metaphase chromosomes were captured with an Axioplan 2 MetaSystems CoolCube1 camera and DAPI-counterstained band images were used for cytogenetic analysis with the Isis software (MetaSystems, Germany).

#### Fluorescent *in situ* hybridization (FISH) of epidermal growth factor receptor

FISH analysis was performed on cytology specimen fixed in methanol or 3.7% formaldehyde prior to hybridization of a dual color probe (Texas Red-labelled EGFR DNA and green fluorescein-labelled CEN-17 PNA) according to the manufacturer's instructions (DAKO). The samples were washed in stringency buffer and stained with DAPI antifade. Axioplan 2 MetaSystems CoolCube1 camera and DAPI-counterstained band images were used for cytogenetic analysis with the Isis software (MetaSystems, Germany).

#### Sphere formation

1000-5000 cells were transferred to plastics without laminin. Sphere formation was monitored and spheres around 100-500 μm were fixated with 4% PFA, embedded in Oct Cryomount (Histolab, Sweden) in cryomolds and frozen in -80°C for later processing.

#### Immunocytochemistry of adherent cells and tumor spheres

Primary antibodies (GFAP monoclonal mouse antibody, Sigma-Aldrich G3893 1:1000; rabbit OLIG2, Chemicon AB9610, 1:300; mouse monoclonal nestin, R&D MAB1259, 1:500; rabbit SOX2, Abcam ab97959; 1:1000; rabbit MAP2, Abcam ab32454, 1:1000; rabbit vimentin, Abcam ab45939, 1:900), were incubated overnight at 4°C. Goat secondary antibodies conjugated to Alexa dyes (Molecular Probes), 1:1000, were added for 1 hr at room temperature and DAPI was used as a nuclear counterstain. For tumor spheres; 10-12 μm thick sections of embedded spheres were cut with a Leica CM3050S Cryostat and mounted on Superfrost™ Plus Slides (Fisher Scientific), which were stored in -20°C until staining and imaging as described above. The Operetta (Perkin Elmer) was used for acquisition of images and the Harmony software for quantification of cells.

#### Differentiation experiment

Cells were seeded in 96-well plates. The day after seeding, media was changed to media with a) EGF (regular stem cell media), b) EGF + FGF (10 ng/ml), c) serum (10%), d) BMP4 (20 ng/ml, Sigma), e) All-trans retinoic acid (ATRA, 10uM, Sigma) and f) no growth factors. Media was changed after 4 days and cells were fixed with 4% PFA after 7 days with EdU added for the last 24 hours. ATRA was added every day.

#### Drug screening assay

Cells were seeded in 96-well plates and the media was changed the next day to media with a) Temozolomide (75μM), b) Etoposide (0.5μM) and c) Vincristine (0.5nM) (Selleckchem). The drugs were dissolved in DMSO and vehicle DMSO controls and untreated controls were included. Media was changed 48 hours after treatment start and cells were fixed with 4% PFA 48 hours later (96 hours treatment).

### DNA methylation profiling

DNA extraction was carried out with the DNeasy Blood & Tissue Kit (Qiagen, Hilden, Germany) according to the protocol provided by the supplier. The DNA was quantified with a Qubit fluorometer (Life Technologies) and 500 ng was used for bisulfite modification using the EZ DNA methylation kit (D5001, Zymo Research, Orange, CA) according to the protocol provided with the modification step according to the recommendations for array processing of the samples. Control PCR reactions were carried out before array analysis to confirm successful modification of the DNA.

The bisulfite-modified DNA was applied to the Infinium HumanMethylation450 BeadChips (Illumina) which determine the methylation levels of more than 450 000 CpG sites. After bisulfite treatment of the DNA samples, the cytosines in the CpG sites were genotyped as C/T polymorphisms according to the manufacturer's protocol. The fluorescence signals were measured from the BeadArrays using an Illumina BeadStation GX scanner. Data analysis and normalization (BMIQ) was done in R with the package ChAMP (Morris et al., 2014). Each CpG site is assigned a score called a “β value”, which corresponds to the ratio between the fluorescence signal of the methylated allele (C) and the sum of the fluorescent signals of the methylated (C) and unmethylated (T) alleles. We set the criteria for MVP calling at significance of *p* < 0.05 (adjusted p-value) and a change in methylation frequency at > 30%. Detection of CNAs was done by using the Conumme R package [38]. *MGMT* methylation was determined using the mgmtstp27 R package (https://github.com/badozor/mgmtstp27/tree/master/trunk/Rpackage).

### *In vivo* experiments

#### Immunocompromised mice

Xenotransplantation of immunocompromised mice, CIEA-NOG (Taconic), 6-8 weeks old females, around 15g, was performed by orthotopically injecting 1*10^5^ tumor cells from three primary cell lines; BPC-A7, BPC-C3 and BPC-C8 into the frontal cortex using a stereotactic frame. The procedure has been described previously [20]. Mice were anesthetized using Isoflurane during transplantation and monitored and weighed every week after surgery. The mice were euthanized at endpoint upon neurological symptoms and/or weight loss.

#### Immunohistochemistry of mouse brain

The brain was removed, fixated in 4% formaldehyde, embedded in paraffin and the tissue was sectioned (5 μm) using a microtome. The sections were de-paraffinized and heat-induced antigen unmasked using citrate buffer pH 6.0 and stained according to Vecta Stain ABC kit (Vector) with rabbit monoclonal hNestin (human specific), ab105389 Abcam, 1:300. H&E was used for histology.

#### Zebrafish

*In vivo* experiments in zebrafish were performed as described in Larsson et al, manuscript in preparation. Briefly, BPC-A7 cells were transduced with RFP lentiviral particles (GenTarget, USA), sorted by FACS (FACS AriaTM II; BD Biosciences, USA) and injected into the ventricles of 2 days post-fertilization (dpf) zebrafish embryo (*Danio rerio*, wildtype AB strain) using a glass micropipette. Confocal imaging of embedded embryos in agarose was performed using a LSM 710 confocal microscope (Zeiss) at the Centre for Cellular Imaging, Sahlgrenska Academy. BPC-A7 and NS cells (control) were injected intranasally into adult zebrafish to establish a survival curve.

#### Study approval

The *in vivo* experiments were approved by the animal ethics committee in Gothenburg (permit number 6-2015 and 10-2015) and were carried out in accordance with the guidelines of the Swedish National Board for Laboratory Animals.

## SUPPLEMENTARY MATERIALS FIGURES AND TABLES



## Abbreviations

GBM: glioblastoma multiforme
CNA: copy number alteration
DIPG: diffuse intrinsic pontine glioma
PNET CNS: Primitive neuroectodermal tumors of the central nervous system
AT/RT: atypical teratoid/rhabdoid tumors
H&E: hematoxylin and eosin
IDH: isocitrate dehydrogenase
MGMT O-6-methylguanine: DNA methyltransferase
EGF: epidermal growth factor
PDGFRA-platelet: derived growth factor receptor
alpha: polypeptide
RB1: retinoblastoma 1
CDKN2A/B cyclin: dependent kinase inhibitor 2A/B
PTEN: phosphatase and tensin homolog
FGF-2: fibroblast growth factor 2
FCM: flow cytometry
OLIG2: oligodendrocyte transcription factor 2
FISH: fluorescence in situ hybridization
SOX2-SRY: (sex determining region Y)-box 2
EdU 5-ethynyl-2: deoxyuridine
GFAP: glial fibrillary acidic protein
BMP-4: bone morphogenic protein 4
ATRA-all: trans retinoic acid
RFP: red fluorescence protein
PFA: paraformaldehyde.

## References

[1] Louis DN, Ohgaki H, Wiestler OD, Cavenee WK, Burger PC, Jouvet A, Scheithauer BW, Kleihues P (2007). The 2007 WHO classification of tumours of the central nervous system. Acta Neuropathol.

[2] Sturm D, Witt H, Hovestadt V, Khuong-Quang DA, Jones DT, Konermann C, Pfaff E, Tonjes M, Sill M, Bender S, Kool M, Zapatka M, Becker N (2012). Hotspot mutations in H3F3A and IDH1 define distinct epigenetic and biological subgroups of glioblastoma. Cancer Cell.

[3] Wu G, Broniscer A, McEachron TA, Lu C, Paugh BS, Becksfort J, Qu C, Ding L, Huether R, Parker M, Zhang J, Gajjar A, Dyer MA (2012). Somatic histone H3 alterations in pediatric diffuse intrinsic pontine gliomas and non-brainstem glioblastomas. Nature genetics.

[4] Schwartzentruber J, Korshunov A, Liu XY, Jones DT, Pfaff E, Jacob K, Sturm D, Fontebasso AM, Quang DA, Tonjes M, Hovestadt V, Albrecht S, Kool M (2012). Driver mutations in histone H3.3 and chromatin remodelling genes in paediatric glioblastoma. Nature.

[5] Brennan CW, Verhaak RG, McKenna A, Campos B, Noushmehr H, Salama SR, Zheng S, Chakravarty D, Sanborn JZ, Berman SH, Beroukhim R, Bernard B, Wu CJ (2013). The somatic genomic landscape of glioblastoma. Cell.

[6] Paugh BS, Qu C, Jones C, Liu Z, Adamowicz-Brice M, Zhang J, Bax DA, Coyle B, Barrow J, Hargrave D, Lowe J, Gajjar A, Zhao W (2010). Integrated molecular genetic profiling of pediatric high-grade gliomas reveals key differences with the adult disease. Journal of clinical oncology.

[7] Hemmati HD, Nakano I, Lazareff JA, Masterman-Smith M, Geschwind DH, Bronner-Fraser M, Kornblum HI (2003). Cancerous stem cells can arise from pediatric brain tumors. Proc Natl Acad Sci U S A.

[8] Johnson RA, Wright KD, Poppleton H, Mohankumar KM, Finkelstein D, Pounds SB, Rand V, Leary SE, White E, Eden C, Hogg T, Northcott P, Mack S (2010). Cross-species genomics matches driver mutations and cell compartments to model ependymoma. Nature.

[9] Monje M, Mitra SS, Freret ME, Raveh TB, Kim J, Masek M, Attema JL, Li G, Haddix T, Edwards MS, Fisher PG, Weissman IL, Rowitch DH (2011). Hedgehog-responsive candidate cell of origin for diffuse intrinsic pontine glioma. Proc Natl Acad Sci U S A.

[10] Singh SK, Clarke ID, Terasaki M, Bonn VE, Hawkins C, Squire J, Dirks PB (2003). Identification of a cancer stem cell in human brain tumors. Cancer research.

[11] Taylor MD, Poppleton H, Fuller C, Su X, Liu Y, Jensen P, Magdaleno S, Dalton J, Calabrese C, Board J, Macdonald T, Rutka J, Guha A (2005). Radial glia cells are candidate stem cells of ependymoma. Cancer Cell.

[12] Bao S, Wu Q, McLendon RE, Hao Y, Shi Q, Hjelmeland AB, Dewhirst MW, Bigner DD, Rich JN (2006). Glioma stem cells promote radioresistance by preferential activation of the DNA damage response. Nature.

[13] Xu J, Margol A, Asgharzadeh S, Erdreich-Epstein A (2015). Pediatric brain tumor cell lines. J Cell Biochem.

[14] Valera ET, de Freitas Cortez MA, de Paula Queiroz RG, de Oliveira FM, Brassesco MS, Jabado N, Faury D, Bobola MS, Machado HR, Scrideli CA, Tone LG (2009). Pediatric glioblastoma cell line shows different patterns of expression of transmembrane ABC transporters after in vitro exposure to vinblastine. Childs Nerv Syst.

[15] Bax DA, Little SE, Gaspar N, Perryman L, Marshall L, Viana-Pereira M, Jones TA, Williams RD, Grigoriadis A, Vassal G, Workman P, Sheer D, Reis RM (2009). Molecular and phenotypic characterisation of paediatric glioma cell lines as models for preclinical drug development. PLoS One.

[16] Hussein D, Punjaruk W, Storer LC, Shaw L, Othman R, Peet A, Miller S, Bandopadhyay G, Heath R, Kumari R, Bowman KJ, Braker P, Rahman R (2011). Pediatric brain tumor cancer stem cells: cell cycle dynamics, DNA repair, and etoposide extrusion. Neuro Oncol.

[17] Ajeawung NF, Maltais R, Jones C, Poirier D, Kamnasaran D (2013). Viability screen on pediatric low grade glioma cell lines unveils a novel anti-cancer drug of the steroid biosynthesis inhibitor family. Cancer Lett.

[18] Lee J, Kotliarova S, Kotliarov Y, Li A, Su Q, Donin NM, Pastorino S, Purow BW, Christopher N, Zhang W, Park JK, Fine HA (2006). Tumor stem cells derived from glioblastomas cultured in bFGF and EGF more closely mirror the phenotype and genotype of primary tumors than do serum-cultured cell lines. Cancer Cell.

[19] Danielsson A, Nemes S, Tisell M, Lannering B, Nordborg C, Sabel M, Carén H (2015). MethPed: a DNA methylation classifier tool for the identification of pediatric brain tumor subtypes. Clin Epigenetics.

[20] Pollard SM, Yoshikawa K, Clarke ID, Danovi D, Stricker S, Russell R, Bayani J, Head R, Lee M, Bernstein M, Squire JA, Smith A, Dirks P (2009). Glioma stem cell lines expanded in adherent culture have tumor-specific phenotypes and are suitable for chemical and genetic screens. Cell Stem Cell.

[21] Pandita A, Aldape KD, Zadeh G, Guha A, James CD (2004). Contrasting in vivo and in vitro fates of glioblastoma cell subpopulations with amplified EGFR. Genes Chromosomes Cancer.

[22] Faria CM, Rutka JT, Smith C, Kongkham P (2011). Epigenetic mechanisms regulating neural development and pediatric brain tumor formation. J Neurosurg Pediatr.

[23] Varley KE, Gertz J, Bowling KM, Parker SL, Reddy TE, Pauli-Behn F, Cross MK, Williams BA, Stamatoyannopoulos JA, Crawford GE, Absher DM, Wold BJ, Myers RM (2013). Dynamic DNA methylation across diverse human cell lines and tissues. Genome Res.

[24] Baysan M, Woolard K, Bozdag S, Riddick G, Kotliarova S, Cam MC, Belova GI, Ahn S, Zhang W, Song H, Walling J, Stevenson H, Meltzer P (2014). Micro-environment causes reversible changes in DNA methylation and mRNA expression profiles in patient-derived glioma stem cells. PLoS One.

[25] Guilhamon P, Butcher LM, Presneau N, Wilson GA, Feber A, Paul DS, Schutte M, Haybaeck J, Keilholz U, Hoffman J, Ross MT, Flanagan AM, Beck S (2014). Assessment of patient-derived tumour xenografts (PDXs) as a discovery tool for cancer epigenomics. Genome Med.

[26] Paul DS, Guilhamon P, Karpathakis A, Butcher LM, Thirlwell C, Feber A, Beck S (2014). Assessment of RainDrop BS-seq as a method for large-scale, targeted bisulfite sequencing. Epigenetics.

[27] Karsy M, Albert L, Tobias ME, Murali R, Jhanwar-Uniyal M (2010). All-trans retinoic acid modulates cancer stem cells of glioblastoma multiforme in an MAPK-dependent manner. Anticancer Res.

[28] Schuldiner M, Eiges R, Eden A, Yanuka O, Itskovitz-Eldor J, Goldstein RS, Benvenisty N (2001). Induced neuronal differentiation of human embryonic stem cells. Brain Res.

[29] Scherer HJ (1938). Structural Development in Gliomas. Am J Cancer.

[30] Eden CJ, Ju B, Murugesan M, Phoenix TN, Nimmervoll B, Tong Y, Ellison DW, Finkelstein D, Wright K, Boulos N, Dapper J, Thiruvenkatam R, Lessman CA (2015). Orthotopic models of pediatric brain tumors in zebrafish. Oncogene.

[31] Singh SK, Hawkins C, Clarke ID, Squire JA, Bayani J, Hide T, Henkelman RM, Cusimano MD, Dirks PB (2004). Identification of human brain tumour initiating cells. Nature.

[32] Vinci L, Ravarino A, Fanos V, Naccarato AG, Senes G, Gerosa C, Bevilacqua G, Faa G, Ambu R (2016). Immunohistochemical markers of neural progenitor cells in the early embryonic human cerebral cortex. Eur J Histochem.

[33] Mack SC, Witt H, Piro RM, Gu L, Zuyderduyn S, Stutz AM, Wang X, Gallo M, Garzia L, Zayne K, Zhang X, Ramaswamy V, Jager N (2014). Epigenomic alterations define lethal CIMP-positive ependymomas of infancy. Nature.

[34] Wiestler B, Capper D, Sill M, Jones DT, Hovestadt V, Sturm D, Koelsche C, Bertoni A, Schweizer L, Korshunov A, Weiss EK, Schliesser MG, Radbruch A (2014). Integrated DNA methylation and copy-number profiling identify three clinically and biologically relevant groups of anaplastic glioma. Acta Neuropathol.

[35] Hennessey PT, Ochs MF, Mydlarz WW, Hsueh W, Cope L, Yu W, Califano JA (2011). Promoter methylation in head and neck squamous cell carcinoma cell lines is significantly different than methylation in primary tumors and xenografts. PLoS One.

[36] Vindelov LL, Christensen IJ, Nissen NI (1983). Standardization of high-resolution flow cytometric DNA analysis by the simultaneous use of chicken and trout red blood cells as internal reference standards. Cytometry.

[37] Meisner LF, Johnson JA (2008). Protocols for cytogenetic studies of human embryonic stem cells. Methods.

[38] Hovestadt V, Zapatka M ((2015)). “conumee: Enhanced copy-number variation analysis using Illumina 450k methylation arrays.”, R package version 0.99.4. http://www.bioconductor.org/packages/release/bioc/html/conumee.html.

