# Supplementary material for: Stem cell cultures derived from pediatric brain tumors accurately model the originating tumors
Source: Oncotarget. 2017 Jan 26;8(12):18626–39. doi: 10.18632/oncotarget.14826 (PMC5386635; doi:10.18632/oncotarget.14826)
Supplement: Supplementary file 1 [file oncotarget-08-18626-s001.pdf]

Stem cell cultures derived from pediatric brain tumors accurately model the originating tumors

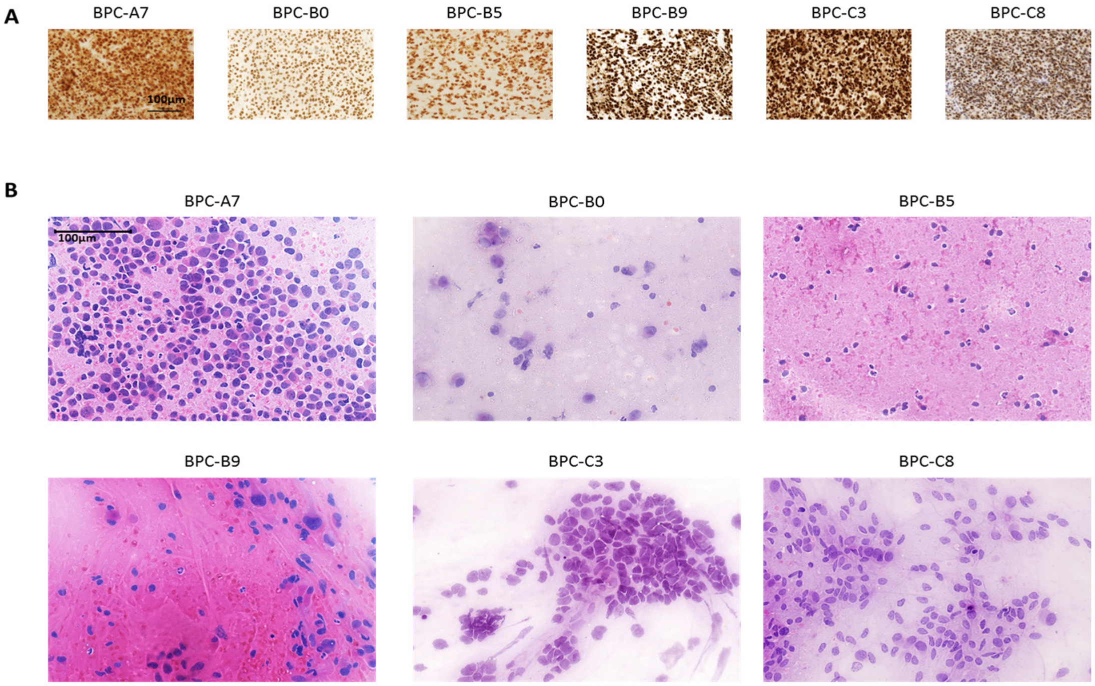

Figure S1. (A) INI1 staining; (B) Hematoxylin and Eosin staining of tumour imprints.

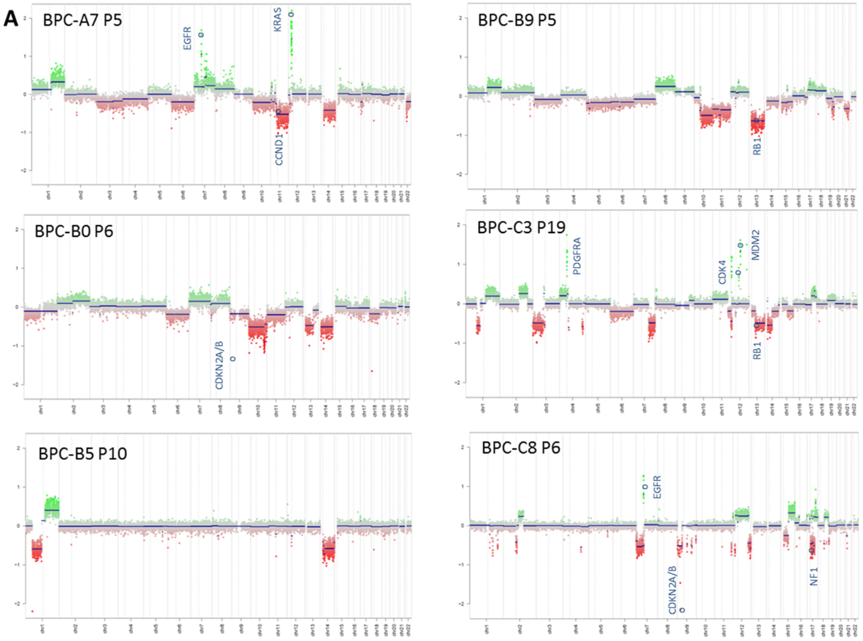

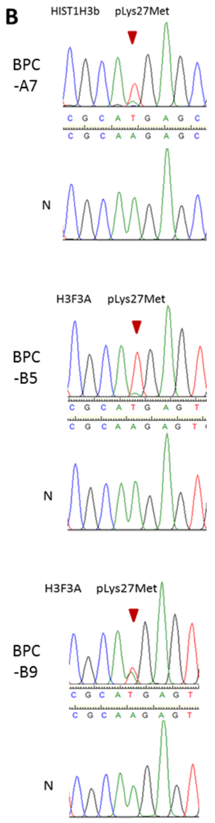

Figure S2. Properties of the cultured cells. (A) Chromosomal profiles (cells between passage 5 and 19) and (B) DNA sequencing of histone mutations of cultured cells (wildtype, N, shown below each cell line).

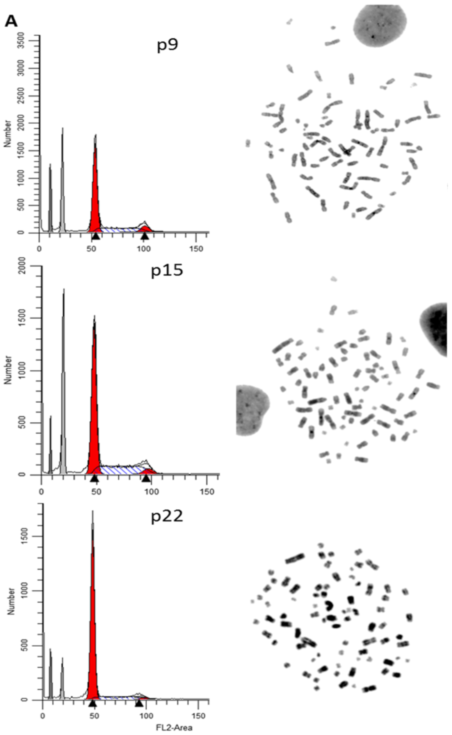

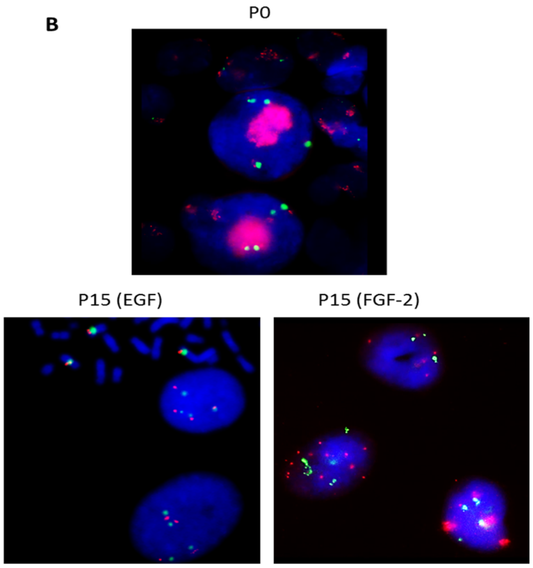

Figure S3. Genomic stability of BPC-A7. (A) The flow cytometry histograms of cell number and FL2-Area (total cell fluorescence) show G1 peak (left red) with DNA index of 1.9, near tetraploid, and G2 peak (right red) through extended passages, grey peaks represent standards with known DNA content (left); modal chromosome numbers of metaphase chromosomes were monitored and found stable over time (right) and (B) *EGFR* amplification status in passage 0 cells (top), after 15 passages in culture (below) – cultured in EGF (left) and in FGF-2 (right). Red, EGFR; green, centrosomal region on chromosome 7.

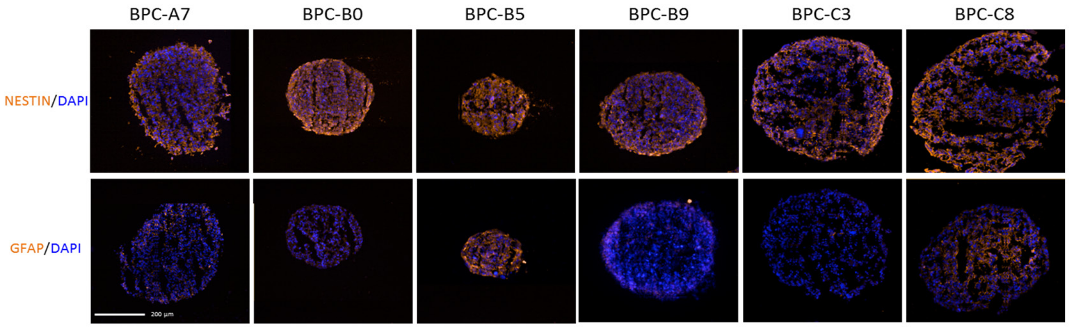

Figure S4. Protein expression in tumour spheres.

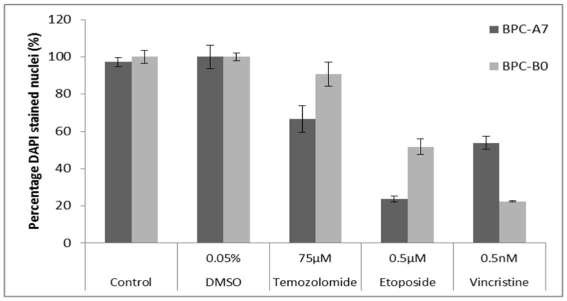

Figure S5. Reproducibility of experiments using the primary cell cultures. BPC-A7 and BPC-B0 show low variation in independent experiments treated with commonly used chemotherapeutic agents; Temozolomide, Etoposide and Vincristine, and DMSO (vehicle) or without treatment (control). The cell lines differ in their response to the treatments. The number of cells is expressed in percentage in relation to the number of cells in the DMSO wells. Error bars denote standard deviation of triplicates.

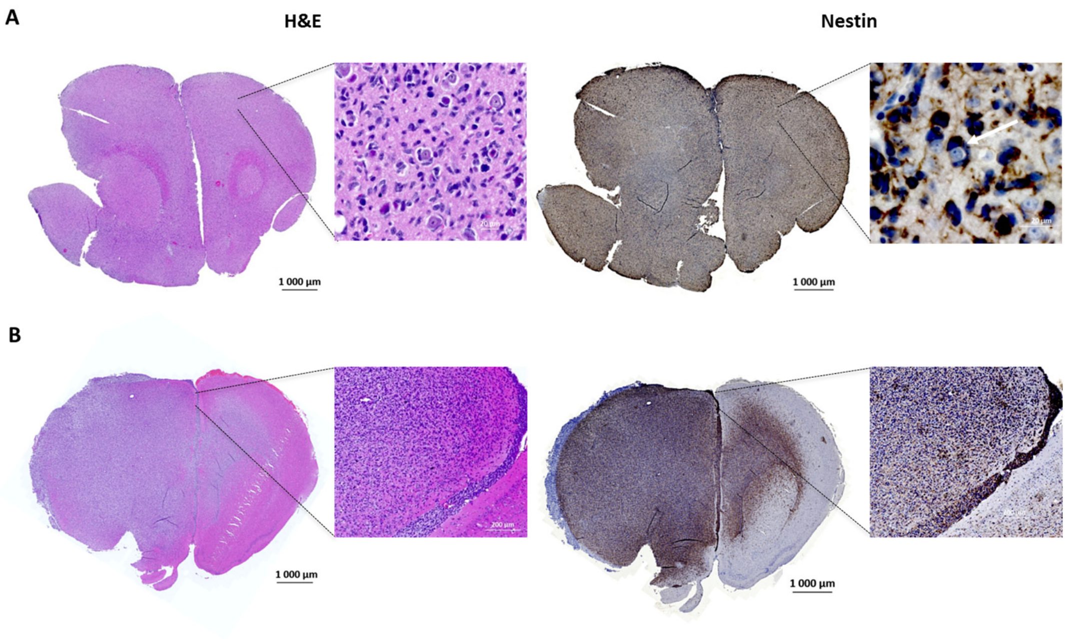

Figure S6. Coronal section of mice brain stained with H&E (left) and human nestin (right). (A) BPC-A7 injected immunodeficient mice demonstrate bilateral invasion of tumour with typical scherer´s structure (tumour cells surrounding nerve cells; see arrow). (B) BPC-C3 injected mice show subpial accumulation of tumour cells. Invasion of white matter into the non-injected hemisphere can also be seen.

Table S1. Patient data.

**Patient-ID**	**Gender**	**Age at diagnosis (years)**	**Location of the tumour**	**Primary or relapse**	**Outcome (years after diagnosis)**
BPC-A7	Male	4.2	Right hemisphere (thalamus)	Primary	DOD (0.9)
BPC-B0	Male	10.4	Right hemisphere (parietal)	Relapse	DOD (2.4)
BPC-B5	Female	12.5	Brain stem	Primary	AWD (1.9)
BPC-B9	Male	6.2	Right hemisphere (thalamus)	Primary	DOD (1.3)
BPC-C3	Female	2.9	Left hemisphere (temporal)	Primary	DOD (0.5)
BPC-C8	Female	11.1	Pons (cerebellopontine angle)	Primary	DOD (0.7)

DOD, dead of disease; AWD, alive with disease
